# Supplementary material for: Preventive behavior of Vietnamese people in response to the COVID-19 pandemic
Source: PLoS One. 2020 Sep 9;15(9):e0238830. doi: 10.1371/journal.pone.0238830 (PMC7480837; doi:10.1371/journal.pone.0238830)
Supplement: S1 File — (DOCX) [file pone.0238830.s001.docx]

**Giới thiệu**

Chính phủ đã thực hiện một số biện pháp để ngăn chặn sự lây lan của coronavirus (COVID-19). Với những câu hỏi dưới đây, chúng tôi muốn tìm hiểu cách bạn thực hiện và áp dụng các hướng dẫn và quy định để chống lại coronavirus. Bạn nên trả lời các câu hỏi này một cách trung thực nhất có thể; điều này giúp chúng tôi đánh giá những biện pháp nào có hiệu quả trong những thời điểm đặc biệt này. Bạn sẽ chỉ mất khoảng 5 - 7 phút để trả lời các câu hỏi.

Việc tham gia khảo sát là tự nguyện, bạn có thể ngừng tiến hành khảo sát bất cứ lúc nào mà không có bất kỳ hậu quả nào. Thông tin của bạn sẽ được lưu trữ ẩn danh và được bảo mật.

Đây là một khảo sát trực tuyến, tự nguyện được khởi xướng bởi một nhóm các nhà nghiên cứu quốc tế đến từ các nước châu Á, châu Phi, Nam Mỹ và châu Âu. Chúng tôi không yêu cầu cung cấp thông tin cá nhân của bạn và dữ liệu sẽ được sử dụng để tìm hiểu tính khả thi và hiệu quả của việc thực hiện các biện pháp phòng ngừa đối với coronavirus ở cấp độ cá nhân.

Khảo sát được thực hiện trong một trang web được bảo mật. Nếu bạn muốn biết thêm thông tin về các nhà nghiên cứu tham gia vào nghiên cứu này và chính sách bảo mật của chúng tôi, bạn có thể xem thêm tại trang web [www.ICPCovid.com](http://www.ICPCovid.com)

Tôi hoàn toàn hiểu nghiên cứu này là gì và tôi đồng ý tham gia. Tất cả thông tin tôi cung cấp có thể được các nhà nghiên cứu sử dụng với mục đích hiểu rõ hơn về coronavirus tại Việt Nam.

Tôi từ chối tham gia nghiên cứu này.

**NGÀY?** _______________________

**THÔNG TIN NHÂN KHẨU HỌC**

**TUỔI** ________________

**GIỚI TÍNH?**

- Nam
- Nữ
- Khác

**QUỐC TỊCH**

- Việt Nam
- Người nước ngoài

**BẠN SỐNG Ở TỈNH/THÀNH PHỐ NÀO?**

- Hà Nội
- Thừa Thiên Huế
- Đà Nẵng
- Quảng Ngãi
- Bình Định
- Khánh Hòa
- Đồng Nai
- TP. Hồ Chí Min
- Cần Thơ
- Tỉnh/TP khác

**TÔN GIÁO**

- Thiên Chúa giáo
- Phật giáo
- Khác
- Không theo tôn giáo nào
- Hồi giáo

**TRÌNH ĐỘ HỌC VẤN CAO NHẤT (ĐÃ TỐT NGHIỆP)**

- Tiểu học
- Trung học cơ sở
- Trung học phổ thông
- Đại học
- Sau đại học

**TÌNH TRẠNG HÔN NHÂN**

- Độc thân
- Đã kết hôn
- Sống thử
- Đã ly hôn
- Góa

**BẠN ĐANG SỐNG CÙNG AI? (CÓ THỂ CÓ NHIỀU CÂU TRẢ LỜI)**

- Bố mẹ
- Vợ/chồng
- Con cái
- Anh chị em hoặc người thân khác
- Bạn bè
- Sống một mình

**CÓ BAO NHIÊU NGƯỜI Ở CÙNG NHÀ VỚI BẠN (KHÔNG KỂ BẠN)? VUI LÒNG GHI SỐ LƯỢNG NGƯỜI TƯƠNG ỨNG VỚI TỪNG NHÓM TUỔI**

- Người trên 70 tuổi: _________________
- Người từ 18 đến 70 tuổi: ________________
- Người từ 12 đến 18 tuổi: ____________________
- Trẻ em dưới 12 tuổi: ________________________________

**BẠN ĐANG SỐNG Ở**

- Vùng nông thôn
- Vùng ngoại ô
- Thị trấn
- Thành phố

**ĐIỀU KIỆN NHÀ Ở CỦA BẠN**

- Nhà hoặc căn hộ có vườn
- Nhà hoặc căn hộ không có vườn
- căn hộ có ban công
- Căn hộ không có ban công
- Một căn phòng
- Túp lều
- Lán trại
- Vô gia cư

**CUỘC SỐNG HẰNG NGÀY TRONG DỊCH DO CORONAVIRUS**

**CÓ BAO NHIÊU NGƯỜI (NGOẠI TRỪ NGƯỜI CÙNG NHÀ) MÀ BẠN ĐÃ NÓI CHUYỆN TRỰC TIẾP NGÀY HÔM QUA (KHÔNG PHẢI QUA ĐIỆN THOẠI, TIN NHẮN, V.V.)?**

(Số lượng)___________________________________________________________

**LẦN CUỐI CÙNG BẠN BẮT TAY, HÔN HOẶC CÓ BẤT KỲ HÌNH THỨC TIẾP XÚC THÂN THỂ NÀO VỚI NGƯỜI KHÁC KHÔNG PHẢI LÀ NGƯỜI CÙNG NHÀ?**

- Hôm nay
- 2 ngày trước đây
- 3-5 ngày trước đây
- Hơn một tuần trước đây
- không tiếp xúc với người khác không ở cùng nhà

**TRONG TUẦN QUA, BẠN CÓ GẶP KHÓ KHĂN TRONG VIỆC CÓ ĐƯỢC THỰC PHẨM?**

- Có
- Không

**LÝ DO QUAN TRỌNG NHẤT KHIẾN BẠN GẶP KHÓ KHĂN TRONG VIỆC CÓ ĐƯỢC THỰC PHẨM TRONG TUẦN TRƯỚC LÀ GÌ?**

- Không có tiền
- Có quá ít thực phẩm trong quầy bán hàng
- Tôi cảm thấy không an toàn khi ra khỏi nhà
- Tôi đã quá yếu để đi ra ngoài

**BẠN HÃY MÔ TẢ MỨC ĐỘ LO LẮNG HAY SỢ HÃI VỀ SỨC KHỎE CỦA MÌNH TRONG TUẦN QUA?**

1= không lo lắng đến 5= cực kỳ lo lắng

**BẠN ĐÃ CHỊU BẤT KỲ HÌNH THỨC BẠO LỰC HOẶC PHÂN BIỆT ĐỐI XỬ VÌ CÁC BIỆN PHÁP CHỐNG DỊCH BỆNH CORONAVIRUS? (CÓ THỂ CÓ NHIỀU CÂU TRẢ LỜI)**

- Bạo lực tại nhà
- Bạo lực ở ngoài
- Phân biệt đối xử vì tình trạng kinh tế / xã hội của tôi
- Phân biệt đối xử vì nhóm dân tộc, chủng tộc hoặc quốc tịch của tôi
- Không bạo lực hoặc không phân biệt đối xử

**LÀM THẾ NÀO ĐỂ BẠN SẮP XẾP VIỆC CHĂM SÓC CON CÁI CỦA BẠN NGÀY HÔM NAY?**

- Một mình chăm sóc ở nhà
- Chăm sóc cùng người nhà ở nhà
- Gửi ở trường/nhà trẻ
- Gửi bạn bè/người quen
- Nhà chú/bác/cô/dì...
- Nhà ông bà
- Ở nhà, với một người giúp việc / bảo mẫu
- Khác
- Không phù hợp (không có trẻ em ở nhà)

**CUỘC SỐNG CÁ NHÂN TRONG DỊCH DO CORONAVIRUS**

**BẠN LÀM GÌ ĐỂ SỐNG?**

- Học sinh
- Không có nghề nghiệp
- Tự làm chủ
- Làm việc cho một người, tổ chức hoặc công ty
- Làm việc nhà nước

**BẠN CÓ PHẢI LÀ NHÂN VIÊN Y TẾ HOẶC SINH VIÊN TRONG NGÀNH Y TẾ?**

- Có
- Không

**ĐIỀU KIỆN LÀM VIỆC HIỆN TẠI CỦA BẠN LÀ GÌ?**

- Làm việc ở nhà
- Làm việc ở không gian mở (chợ, cửa hàng, ven đường...)
- Làm một mình trong phòng (văn phòng...)
- Làm trong phòng với một vài người khác (văn phòng...)
- Không áp dụng (thất nghiệp hoặc sinh viên)

**BẠN THƯỜNG ĐI HỌC HOẶC ĐI LÀM BAO NHIÊU NGÀY TRONG TUẦN?** (Số ngày)________________

**BẠN ĐÃ ĐI HỌC HOẶC ĐI LÀM BAO NHIÊU NGÀY VÀO TUẦN TRƯỚC?** (Số ngày)____________________

**BẠN CÓ LÀM VIỆC Ở NHÀ NGÀY HÔM NAY KHÔNG?**

- Có
- Không
- Không áp dụng (thất nghiệp hoặc sinh viên)

**CÁC BIỆN PHÁP BẢO VỆ CÁ NHÂN**

*(Những biện pháp bảo vệ mà bạn đã sử dụng trong tuần qua?)*

**TÔI TUÂN THỦ QUY TẮC KHOẢNG CÁCH TỪ 1,5-2M TRONG XÃ HỘI**

- Có
- Không

**TÔI ĐEO KHẨU TRANG KHI RA NGOÀI**

- Có
- Không

**KHI TÔI HO HOẶC HẮT HƠI, TÔI CHE MIỆNG VÀ MŨI BẰNG KHĂN GIẤY HOẶC HO VÀO KHUỶU TAY**

- Có
- Không

**KHI TÔI HO HOẶC HẮT HƠI, TÔI LUÔN RỬA / SÁT KHUẨN TAY NGAY SAU ĐÓ**

- Có
- Không

**TÔI ĐO NHIỆT ĐỘ CƠ THỂ ÍT NHẤT HAI LẦN MỘT TUẦN**

- Có
- Không

**TÔI RỬA TAY BẰNG XÀ PHÒNG VÀ NƯỚC THƯỜNG XUYÊN TRONG NGÀY**

- Có
- Không

**TÔI SỬ DỤNG CHẤT KHỬ TRÙNG TAY THƯỜNG XUYÊN TRONG NGÀY**

- Có
- Không

**TÔI TRÁNH CHẠM VÀO MẶT (MẮT, MŨI VÀ MIỆNG)**

- Có
- Không

**TÔI KHỬ TRÙNG ĐIỆN THOẠI BẤT CỨ KHI NÀO TÔI TRỞ VỀ NHÀ**

- Có
- Không

**BẠN GẶP KHÓ KHĂN NHƯ THẾ NÀO KHI TUÂN THEO CÁC BIỆN PHÁP BẢO VỆ Ở NHÀ NHIỀU NHẤT CÓ THỂ?**

- Trên thang điểm 1 (= không khó khăn chút nào) đến 5 (=cực kỳ khó khăn)

**CÁC BIỆN PHÁP PHÒNG NGỪA CỘNG ĐỒNG ĐỐI VỚI CORONAVIRUS**

**BẠN RỬA TAY HOẶC SỬ DỤNG NƯỚC SÁT KHUẨN KHOẢNG BAO NHIÊU LẦN TRONG NGÀY HÔM QUA?** (Số lượng)_________

**BẠN CÓ THAM GIA MỘT CUỘC HỌP HOẶC TẬP HỢP VỚI HƠN 10 NGƯỜI TRONG BẢY NGÀY QUA KHÔNG?**

- Có
- Không

**BẠN CÓ ĐI ĐẾN MỘT NHÀ HÀNG, QUÁN BAR, CÂU LẠC BỘ, NƠI KHIÊU VŨ, TIỆC TÙNG, HOẶC BUỔI HÒA NHẠC TRONG BẢY NGÀY QUA?**

- Có
- Không

**BẠN CÓ ĐI ĐẾN MỘT BUỔI HỌP MẶT TÔN GIÁO TRONG BẢY NGÀY QUA?**

- Có
- Không

**BẠN ĐÃ THAM DỰ MỘT ĐÁM TANG TRONG 7 NGÀY QUA?**

- Có
- Không

**BẠN ĐÃ Ở TRONG MỘT CHIẾC XE HOẶC XE BUÝT CÓ HƠN 5 NGƯỜI TRONG BẢY NGÀY QUA?**

- Có
- Không

**BẠN ĐÃ Ở TRONG MỘT PHÒNG TẬP THỂ DỤC CÔNG CỘNG (PHÒNG GYM) TRONG 7 NGÀY QUA?**

- Có
- Không

**BẠN ĐÃ ĐI ĐẾN MỘT TIỆM LÀM ĐẸP, MÁT-XA, SPA, LÀM TÓC HOẶC LÀM MÓNG TRONG 7 NGÀY QUA?**

- Có
- Không

**BẠN CÓ ĐI CHỢ TRONG 7 NGÀY QUA KHÔNG?**

- Có
- Không

**BẠN ĐÃ SỬ DỤNG ĐĨA HOẶC THÌA CHUNG KHI ĂN CÙNG VỚI CÁC THÀNH VIÊN TRONG GIA ĐÌNH TRONG 7 NGÀY QUA?**

- Có
- Không

**BẠN ĐÃ SỬ DỤNG ĐĨA HOẶC THÌA CHUNG KHI ĂN CÙNG VỚI NHỮNG NGƯỜI KHÔNG PHẢI LÀ THÀNH VIÊN TRONG GIA ĐÌNH**

**TRONG 7 NGÀY QUA?**

- Có
- Không

**BẠN CÓ ĐI DU LỊCH TRONG 7 NGÀY QUA?**

- Vâng, tôi đi du lịch ở tỉnh khác
- Vâng, tôi đi du lịch nước ngoài
- Không du lịch

**TRONG TUẦN QUA, BẠN ĐÃ LO LẮNG HAY SỢ HÃI THẾ NÀO VỀ SỨC KHỎE CỦA NHỮNG NGƯỜI THÂN YÊU?**

- Trên thang điểm 1 = không lo lắng đến 5 = cực kỳ lo lắng

**TRÊN THANG ĐIỂM TỪ 1 ĐẾN 10, BẠN CÓ THỂ CHỈ RA MỨC ĐỘ MÀ MỌI NGƯỜI XUNG QUANH BẠN THỰC TẾ ĐÃ ĐIỀU CHỈNH HÀNH VI CỦA HỌ THEO CÁC KHUYẾN NGHỊ CỦA CHÍNH PHỦ KHÔNG?**

- (*1=* *không điều chỉnh, 10=* *điều chỉnh rất mạnh*).

**BẠN THƯỜNG TIẾP CẬN THÔNG TIN VỀ DỊCH COVID-19 TỪ NHỮNG NGUỒN NÀO?**

- Truyền hình, truyền thanh (tivi, loa, đài...)
- Trang web của Bộ Y tế
- Báo điện tử
- Mạng xã hội
- Người thân, bạn bè
- Khác....

**CÂU HỎI LIÊN QUAN ĐẾN SỨC KHỎE CÁ NHÂN**

**BẠN ĐÃ ĂN NHIỀU HƠN THỰC PHẨM LÀNH MẠNH NHƯ TRÁI CÂY VÀ RAU QUẢ KỂ TỪ KHI DỊCH CORONAVIRUS BẮT ĐẦU?**

- Có
- Không

**BẠN ĐÃ VÀ ĐANG UỐNG NHIỀU VIÊN VITAMIN HƠN?**

- Có
- Không

**BẠN CÓ CÁC TRIỆU CHỨNG GIỐNG NHƯ CÚM TRONG 7 NGÀY QUA (HO HOẶC ĐAU HỌNG, KHÓ THỞ, ĐAU ĐẦU, ĐAU CƠ THỂ, SỐT)?**

- Có
- Không
- Không biết

**BẠN ĐÃ CÓ NHỮNG TRIỆU CHỨNG GIỐNG CÚM BAO NHIÊU NGÀY?**

Số ngày __________

**NHỮNG TRIỆU CHỨNG NÀY VẪN CÒN?**

- Có
- Không

**CÓ AI TRONG SỐ NHỮNG NGƯỜI Ở CÙNG NHÀ VỚI BẠN CÓ CÁC TRIỆU CHỨNG GIỐNG NHƯ CÚM TRONG 7 NGÀY QUA KHÔNG?**

- Có
- Không
- Không biết

**BẠN CÓ HÚT THUỐC KHÔNG?**

- Có
- Không

**BẠN CÓ MẮC MỘT BỆNH LÝ NỀN (VÍ DỤ: BỆNH TIM, HEN SUYỄN, TIỂU ĐƯỜNG, TĂNG HUYẾT ÁP, UNG THƯ, HIV, BỆNH LAO, V.V.) KHÔNG?**

- Có
- Không
- Không biết

**CÁC HOẠT ĐỘNG MÀ BẠN DÀNH THỜI GIAN RIÊNG VỚI TRẺ EM VÀO THỜI ĐIỂM NÀY (Ở NHÀ, GIÃN CÁCH XÃ HỘI):**

- Kể chuyện, trao đổi điều trẻ em thích, đọc sách hoặc xem những bức ảnh
- Đi bộ - đi ở ngoài trời hoặc xung quanh nhà
- Tập thể dục cùng với nhau trên điệu nhạc yêu thích
- Làm việc nhà cùng nhau và kiến tạo trò chơi thông qua việc quét nhà và nấu ăn
- Giúp đỡ con trong việc học
- Không áp dụng (không có trẻ em dưới 19 tuổi)
- Không dành thời gian cho trẻ em
- Khác (ghi rõ)

**CÁC HÌNH THỨC VẬN ĐỘNG CƠ THỂ CỦA BẠN TRONG MÙA DỊCH COVID-19 NÀY:**

- Đi bộ - đi ở ngoài trời hoặc xung quanh nhà
- Tập thể dục cùng với nhau trên điệu nhạc yêu thích
- Nhún nhảy theo nhạc
- Tham gia lớp tập thể dục trực tuyến
- Đạp xe đạp
- Khác (ghi rõ)

Cảm ơn bạn rất nhiều vì sự tham gia của bạn. Chúng tôi muốn mời bạn tham gia vào một cuộc khảo sát khác trong hai tuần tới. Sự tham gia là tự nguyện và không bắt buộc.
