# Supplementary material for: Preventive behavior of Vietnamese people in response to the COVID-19 pandemic
Source: PLoS One. 2020 Sep 9;15(9):e0238830. doi: 10.1371/journal.pone.0238830 (PMC7480837; doi:10.1371/journal.pone.0238830)
Supplement: Attachment — Submitted filename: Review comment _AnswerVVT.docx [file pone.0238830.s002.docx]

| # | Comments |  |
| --- | --- | --- |
| A. Additional Editor Comments | | |
| 1 | Your abstract concludes with the statement that preventive behavior was "excellent" and then states that this behavior explains the low number of infections and mortality. The first statement presents the appearance of bias and should be rephrased. The implication that these behaviors led to low infection rates and zero mortality cannot be supported based on your evidence alone and should be removed. | Thank you for this comment. We have rewritten the abstract and removed our statement that was the cause of the low numbers of infection and mortality. We now state that the behavioral findings are consistent with the low levels of infection in Vietnam.  Please see in line 39-41 in page 2. |
| 2 | In your sample, healthcare workers appear to be heavily over-represented. One could reasonably consider this to be a sample of healthcare worker behaviors rather than a general population sample. Please consider revising your analysis to only include healthcare workers. While not your original intent, this might be a more meaningful analysis. | We agree this is a key limitation of our study. Our snowball sampling was initiated via social networks linked with medical universities and therefore a majority of participants were health care workers. We mention this in the discussion as a limitation of the study. However, a relatively large number of respondents were also non-health care workers (502). Therefore, we propose to keep the total number of 2,175 respondents in our analysis. Moreover, given the low numbers of COVID-19 infections in Vietnam it is likely that the level of adherence to preventive measures was also good in non-health care workers. Please see Line 290-299 at page 17 of our revised paper. |
| 3 | In your discussion, you state that "positive attitudes" indicate most people believe in government requirements. Your data does not support this statement, as you only measured reported compliance and did not ask about attitudes toward the requirements. If information about attitudes toward requirements was obtained, it should be presented in the results section clearly. | We deleted that "positive attitudes" indicate most people believe in government requirements. Indeed, we only assessed adherence to preventive measures. |
| B. Reviewer #1 | | |
| 1 | Although the participants resided in 55/63 provinces of Vietnam, there were only about 40 participants in a province. The sample size was not estimated. It is not sure whether the sampling is representative. | 1054 (48.5%) of the respondents lived in major municipalities (Ha No, Ho Chi Minh City, Hai Phong, Can Tho, and Da Nang) and 1121 (51.5%) in smaller urban or rural areas. We did not however collect information per province. Indeed, it may well be that certain provinces were under represented in our study sample. We now mention this in the discussion  Our sample size was not estimated because we opted for convenient sampling.  Moreover because of the snow-ball sampling starting mainly from medical students and health care workers, our sample is not representative of the general population of Vietnam. Females were indeed overrepresented. We mention this as a limitation of our study (line 290-299 in page 17). Based on the results of our multiple linear regression analysis investigating factors associated with adherence to the COVID-19 preventive measures sex was not a significant determinant of adherence. |
| 2 | Due to the snow-ball sampling, there were apparently unbalanced characteristics of study participants investigated, such as the number of female was larger than that of male. These might affect the results of the study. |  |
| 3 | The author should provide the questionnaire as a supplementary file to show more details of the survey. | Please see our questionnaires in the appendix of the manuscript |
| 4 | The definitions of some abbreviations or parameters should be given at their first used, such as CI and β in the Abstract section. | Thank you for reminding us to follow this standard practice. We now replace all abbreviations at their first use, including β with “beta” and CI with “Confidence interval”  See line 35 at page 2. |
| C. Reviewer #2 | | |
| Abstract | |  |
| 1 | Page 2, line 22: Please spell out Coronavirus Disease 2019 (COVID-19) in its first use. | We have replaced “COVID-19” with “Coronavirus Disease 2019” in its first use. (line 22 at page 2) |
| 2 | Page 2, line 24: Please consider editing as "...survey was administered..." or "...data were collected via an online questionnaire..." or something similar (versus organized) | We replaced “organized” with “administered”  See Line 24 at page 2. |
| 3 | Page 2, line 25: Please briefly describe the preventative behaviors referenced here. | Abstracts in Plos should not exceed 300 words, so it was not possible to list all preventive behaviours. We now state *“The questionnaire assessed personal preventive behavior (such as physical distancing, wearing a face mask, cough etiquette, regular handwashing and using an alcohol hand sanitizer, body temperature check, and disinfecting mobile phones) and community preventive behavior (such as avoiding meetings, large gatherings, going to the market, avoiding travel in a vehicle/bus with more than 10 persons, and not traveling outside of the local area during the lockdown).“*  All measures are explained in the method section (page 8, line 117-130, page 6-7) |
| 4 | Page 2, line 26: Please provide the mean (SD) age. | This is now added in Line 31-32 page 2. |
| 5 | Page 2, line 27: How many items were included, and how were these items scored? This is needed to be able to interpret these scores, and put these findings in context. Please indicate whether adherence to personal and community measures were considered together in analyses. | **Personal preventive** measures were assessed by using 9 questions  **Community preventive** measures were assessed with 11 questions.  Self-reported adherence with each preventive measure was given a score of 1, with 0 for non-adherence.  The total adherence score was calculated by summing both personal and community prevention strategies (11+9=20 questions). Adherence to personal preventive measures is described in table 3 (Line 194 at page 11); adherence to community preventive measures is described in table 4 (Line 205 at page 12).  We now state in the abstract “*A total adherence score was calculated by summing the scores of the 9 personal and the 11 community prevention questions*.”  See line 29-31 at page 2. |
| 6 | Page 2, line 34: Please identify this low number based on the Vietnamese population. | We added “Our findings are consistent with the epidemiology of COVID-19 in Vietnam, where there have been few infections and no recorded deaths up to the first week of July 2020”, please see in line 41-42, page 2.  As of May 5^th^, 2020, Vietnam had totaled 271 confirmed cases with zero deaths over Vietnamese population of 97,141,003 (10 April 2020). <https://en.wikipedia.org/wiki/Demographics_of_Vietnam> |
| Introduction | |  |
| 7 | Page 3, line 38: Please begin by identifying the virus SARS-CoV-2. | We started our paper with:  *Coronaviruses (CoVs) are a group of viruses which co-infect humans and other animals of the vertebrates. CoV infections affect humans, cattle, birds, bats and many other wild animals in the respiratory, gastrointestinal, liver and central nervous systems (1).* |
| 8 | Page 3, line 50: Were there any mandatory stay-at-home/quarantine orders in effect at this time, or was isolation at this point strictly among those who were infected? | All people were required to stay at home, and instructed that they should only go out in case of necessity  Please also see Line 55-59 at page 3 for a further reference |
| 9 | Page 4, line 61: Please provide the scientific names of these infectious outbreaks. | We now added the scientific names. Thank you  See in Line 69-71 at page 4  Severe Acute Respiratory Syndrome (SARS), Swine Flu (A/H1N1pdm09 virus, also known as ‘H1N1’), and Avian influenza (Avian influenza virus subtypes A) |
| 10 | Page 4, line 74: Please specify what types of effects were assessed (changes in routine activities, behavioral, psychological, physical)? | We assessed routine activities such as difficulty in obtaining food, protective behavior (eating more vegetables and consuming more vitamins), physical activities (what exercise people did during the social distancing period).  Please see in line 136-139 page 7. |
| Methods | |  |
| 11 | Page 5, line 85: How was consent indicated on this online assessment? Were the participants compensated in any way? | A full description of the study was provided in the Vietnamese language on the [www.icpcovid.com](http://www.icpcovid.com) website, and all participants had to click on: *“I fully understand what this study is about, and I consent to participate. All the information I provide can be used by researchers to better understand coronavirus disease”* before submitting the responses.  Participation in the survey was voluntary and without compensation for participants.  Please see Questionnaires in the appendix for your further reference. |
| 12 | Page 5, lines 97-98: Please provide details of the categories of these socio-demographic variables. | We added the socio-demographic variables in the conceptual framework.  Please see Figure 1 in Line 95 at page 5. |
| 13 | Page 5, line 103: Please specify that this is adherence to personal and community measures. | We confirm that overall adherence was a combination of both personal and community measures. |
| 14 | Page 6, line 122: This is somewhat unclear- how is perceived adaptation of the community defined/conceptualized in this context? | “Perceived adaptation of the community is” was assessed by asking the participants how well they perceived that the community adapted to comply with the government recommendations to fight COVID-19. It was a self-assessment, reported as a Likert score.  See our revision in Line 140-143 at page 7 |
| 15 | Page 6, lines 119-124: Were these relationships to adherence still examined or strictly controlled for as covariates? These are important factors that could have a significant impact on adherence. Please clarify their role in analyses. | Thank you. The relationship between cofactors is shown in a conceptual frame work (Fig 1)  Please see Figure 1 in Line 95 at page 5. |
| 16 | Page 7, line 133: Please clarify whether this was one multiple linear regression or a series of models, and specify how covariates were included. Also, please identify all of the independent variables here. | We used a multivariate linear regression model to identify factors associated with adherence to the COVID-19 preventive measures.  **Dependent variable:** adherence scores. We used square transformation to convert the scores into an approximate normal distribution.  **Covariates:** First, age and gender were included in the model to adjust for other covariates. After that, all factors were kept in the model if they had a statistically significant relationship to adherence at p<0.05.  Please see this revision in data analysis section (page 8, line 154-159) |
| Results | |  |
| 17 | Page 7, lines 140-141: How is there overlap, as these percentages exceed 100%? Perhaps identifying the specific categories and their associated percentages would be helpful. Also, these percentages do not correspond with what is included in Table 1 on page 8. | In Vietnam as in China, we use the term “municipalities” This is usually a major city that is a semi-autonomous administrative division having corporate status and powers of self-government as granted by national and regional laws to which it is subordinate. There were 5 cities mentioned in line 164 and 165 (page 8). All 5 cities are classified in the “urban” group that includes other, smaller cities in Vietnam. Hence, the proportion of “urban” (65.8%) included the “municipalities” (48.5%).  We now state *“The participants resided in 55/63 provinces of Vietnam: 1054 (48.5%) lived in major municipalities (Ha No, Ho Chi Minh City, Hai Phong, Can Tho, and Da Nang) and 1121 (51.5%) lived in smaller urban or rural areas”.* Please see in line 163 – 165, page 8.  Thank you for your comment. |
| 18 | Page 7, Table 1: As mentioned earlier, please include all categories. Also, please reformat the table so that the categories align better with the descriptors/category levels in the middle columns. | We now added all categories as requested.  Please see Table 1 in Line 169 at page 9. |
| 19 | Page 8, Table 1, Health Professional/Student category: Why were health professional and student combined? It is also not specified that these are medical students. This is a very high proportion of the total sample. Please provide explanation regarding whether sampling was targeted to health care professionals, or was this a community sample? Also, there is a possibility that health professional and medical students, despite their training, may have different experiences in their response to the pandemic. Please consider conducting the analyses with these groups examined separately. | Thank you for your comment. Indeed students were medical students. We now specify this in the table and text.  As we now explain in the methods that the study emanated from a medical-university. Links to the survey were send to colleagues, graduates and students, who were then asked to recommend the survey to people in their own networks.  We also analyzed the group of medical students separately from health professionals and did not observe any significant difference in behavior.  We mention the over representation of health care workers as a limitation of the study (line 290-299 in page 17) |
| 20 | Page 8, Table 1, Currently living with: Is living with spouse or partner a category? Again, please include all categories of all of the variables included in the analyses. | We now added all categories.  Please see Table 1 in Line 169 at page 9-10. |
| 21 | Page 8, Table 1, Smoking/Eating more healthy food/Taking more vitamin tablets: Is this within the past week? Please specify. | We collected data within the past 7 days during our social distancing measures in Vietnam.  We now specify in Table 1, (line 169 in page 9-10)  “Currently smoking”  “Eating more healthy food since the COVID epidemic”  “Taking more vitamin tablets since the COVID-19 epidemic” |
| 22 | Page 8, line 152: These items need to be specified within the Measures section. It is not until this point that the reader is aware that these factors were assessed. Please confirm that all variables in the study are defined/described in the Measures section. | We now mention in the Measures section *“Difficulties in obtaining food” and “Experienced violence or discrimination during the confinement”, please see in line 176-178 in page 10 and* Figure 1 (line 95, page 5).  And also see Table 2 in Line 181 at page 10. |
| 23 | Page 8, line 154: Please initially report this coding scheme in the Methods/Measures section. | We added this information about study covariates: “*Possible difficulties with daily life activities during the previous week (such as working from home, access to food, access to medication for respondents with underlying chronic conditions, violence/discrimination as a consequence of the lockdown measures) were also assessed using yes/no questions.”*  See Line 136-139 at page 7. Also see in Figure 1 for futher information. |
| 24 | Page 9, Table 2: As with Table 1, please reformat, as it is unclear how these variables are categorized, and the alignment seems off. Please address with all tables | We have reformatted the table to improve the clarity for all categories (see the revised Table 1 and Table 2). |
| 25 | Page 9, line 163: Please identify the percentage of those who responded 4 or 5 (or moderate or more?). Please ensure that all of the response options are listed in the Measures section. | We have re-phrased this section to describe more clearly how we measured personal and community adherence to the preventive measures, and how we assessed the level of difficulty experienced in attaining the nominated level of adherences to personal preventive measures.  Adherence assessment was shown at first heading, followed by difficulty level See lines 118-130 in page 6-7.  We have added percentages for those who reported a score of ≥3 indicating that they had moderate to high levels of fear and worry about their own health (30.0%) and their relatives (42.3%) and listed all options in the measures section. Please see in line 134-135 in page 7.  Also see in Table 2 (line 181, page 10). |
| 26 | Page 9, line 178: This categorization should be initially introduced in the Measures section. | We now added the category of perceived community adaptation in response to your comments in the Measurement section  Please see more in Lines 125 – 130 on page 7. |
| 27 | Page 11: If this corresponds with the journal's standards, please include the B and p-value in text as well, to provide the reader with information about the direction and strength of the associations. | We now added beta and p value in text on all significant variables (from line 214 to 219 in page 13) |
| 28 | Page 190: Please provide more details regarding the "official sources of COVID-19 information" factor. All of the factors need to be clearly defined/identified in the Measures section, as the study examines many variables. | We now mention in the Measures section “The question was also asked about the source of information about COVID-19: options that were proposed included: Regarding information from “National television”, “Radio”, “Vietnamese Ministry of Health website”, and the “WHO website”.  See line 143-146 in page 7. |
| Discussion | |  |
| 29 | Page 13, line 215: Please include all of these rating categories in the Measures section. | We now mentioned this aspect in table 2 (line 181, page 10) and also in the methodology (line 111, page 6). |
| 30 | Page 13, line 219: Please add 'and' ("...such as wearing a mask and washing hands frequently...") | Thank you, we added the “ “  Please see in line 249-250 in page 15. |
| 31 | Page 14, bottom of page: Did the authors examine whether there were other demographics associated with adherence? | In our analysis, we only aimed to compare adherence between residents who live in municipalities or not in municipalities.  Please see this information in table 5 on page 14, line 223. |
| 32 | Page 15: Please provide additional interpretation of the main findings of the study (worries about health, difficulty staying at home). | We have discussed these findings regarding “worries about health” in lines 244-248, page 15.  Regarding the finding of “difficulty staying at home” was not applicable in our data analysis. |
| 33 | Page 15, line 262: Can the authors identify the proportion of those of lower socioeconomic status having internet access in Vietnam, or more details about this in general? It is also important to comment on the possibility that certain demographic groups may not have comparable access to means by which to stay safe (social distancing, ready access to clean or disposable masks and hand sanitizer). | In 2018, the internet penetration rate reached 57 percent among Vietnamese and 66% in 2019.(<https://www.statista.com/statistics/369732/internet-users-vietnam/>; https://vnetwork.vn/en/news/cac-so-lieu-thong-ke-internet-viet-nam-2019).  A new study in July 2020 has found Vietnam to be the 10th cheapest country in the world to purchase mobile data (https://e.vnexpress.net/news/news/vietnam-among-world-s-cheapest-mobile-data-markets-report-4105527.html).  This shows internet accessibility of those of lower socioeconomic is increasing and for most, access is not particularly difficult.  However, we agree that it is possible that certain groups not participating in the survey may have had more difficulties with physical distancing and access to face masks and hand sanitizers. |
|  |  |  |
